# Association of serum alkaline phosphatase and depression in US adults: a population-based cross-sectional study

**DOI:** 10.3389/fpsyt.2023.1131105

**Published:** 2023-05-17

**Authors:** Yujiang Liang, Yafei Mao, Weizhong Liang, Liping Liang, Min Suo, Juan Xue, Hui Yang

**Affiliations:** ^1^Department of Laboratory Medicine, Fengfeng General Hospital of North China Medical & Health Group, Han Dan, Hebei, China; ^2^Department of Laboratory Medicine, The First Hospital of Hebei Medical University, Shijiazhuang, Hebei, China; ^3^Hebei Medical University, Shijiazhuang, Hebei, China; ^4^Department of Orthopaedics, Fengfeng General Hospital of North China Medical & Health Group, Han Dan, Hebei, China

**Keywords:** alkaline phosphatase, depression, national health and nutrition examination, mental health, diabetes

## Abstract

**Background:**

Depression, a serious public health disorder, is increasingly prevalent worldwide. An association between alkaline phosphatase (ALP) and neurological disorders has been reported. However, data on ALP and depression risk are scarce, which warrants attention.

**Methods:**

We assessed the association between ALP and risk of depression in adults from the 2007–2014 National Health and Nutrition Examination Survey (NHANES). Depression was assessed using the Patient Health Questionnaire-9. Univariate and multivariate logistic regression were used to assess the association between ALP and risk of depression, and subgroup analyses were also performed.

**Results:**

A total of 17,485 participants were included. The prevalence of depression was 9.3% (1,631/17,485) and ALP was significantly associated with the risk of depression when ALP was a categorical variable (quadratic or categorized by 79 U/L) in a multivariate logistic regression model after adjusting for confounding factors (≥79 U/L vs. <79 U/L, adjusted OR, 1.15; 95%CI, 1.02–1.29). Each 1-unit increase in ALP (log_2_) was associated with a 20% increase in depression prevalence (adjusted OR, 1.20; 95%CI, 1.06–1.36) when ALP was used as a continuous variable. Subgroup analysis showed that ALP was positively associated with the risk of depression with different characteristics.

**Conclusion:**

Our findings suggest that higher alkaline phosphatase levels, even within the normal range, are significantly associated with a higher risk of depression in US adults. Such findings require further prospective studies to provide more evidence.

## 1. Introduction

Depression is a clinically significant and growing public health problem (1). Major depressive disorder (MDD) has become a common disorder that severely limits psychosocial functioning and reduces quality of life. WHO ranked MDD as the third leading cause of the global burden of disease and projected that the disorder will rank first by 2030 (2). The burden of depression is exacerbated by the COVID-19 pandemic blockade, social isolation, economic stress, and other effects (3), and severe depression increases the risk of other illnesses and suicide. In the United States, the lifetime risk of major depressive episode is now estimated to be close to 30% (1). Depression, characterized by a very low mood in all aspects of life and an inability to experience a sense of joy, is one of the most common and distressing disorders worldwide (4). Although social and cultural factors (e.g., socioeconomic status) may play a role in MDD, genomic and other underlying biological factors ultimately drive this condition (5). Depression has been previously reported as a major cause of morbidity and poor quality of life in patients with cardiovascular disease (CVD) (6), diabetes (7), arterial stiffness (AS) (8) and osteoporosis (9) and one study (10) showed a significant association between MDD and increased mortality in all settings and populations evaluated.

Serum alkaline phosphatase (ALP), a specific enzyme existing in almost all organisms (11), is a complex phenotype influenced by genetic and environmental factors (12). Tissue non-specific alkaline phosphatase (TNAP) is expressed in almost all tissues, especially in liver, kidney and bone (13), and is also present in endothelial cells, neuronal membranes, and synaptic contacts of the brain (14). ALP screening is useful to determine the presence of liver disease or liver damage and bone disease, among others. It has been shown that ALP is a reliable marker of cardiovascular events and mortality, such as stroke in hypertensive patients (15) and spontaneous cerebral hemorrhage (16), and also correlates with inflammatory conditions such as knee osteoarthritis (9). A study by S. Graser et al. (17) showed that the expression of TNAP varies with the developmental stage of the brain and that alkaline phosphatase activity is located in the primary visual, auditory and somatosensory cortices in layer 4 of the thalamocortical innervation next to nerve cells, and also varies according to sensory experience. Indicating that TNAP is associated with the nervous system, increased ALP has been associated with cognitive impairment and psychiatric disorders during neocoronary pneumonia in previous reports (18). Depression is the most common psychiatric symptom, and evidence on the relationship between ALP and risk of depression is limited and somewhat confounding (19–22), for example, alkaline phosphatase was not elevated in depressed patients (*N* = 19) compared to healthy controls (19), whereas Petronijevic et al. (20) found that the depressed group of premenopausal women (*N* = 73) compared to the control group had elevated ALP levels, and a recent study (21) found that a higher risk of depression was associated with elevated ALP in women, but not in men. In addition, these existing studies suffer from several limitations, such as small sample sizes and inadequate adjustment for important covariates (e.g., some important blood biochemical indicators and associated comorbidities). In addition, it remains unclear whether ethnicity/race, smoking status, comorbid diabetes, and hypertension can modify the correlation of concern.

Hence, we extracted data on depression from the National Health and Nutrition Examination Survey (NHANES) from 2007 to 2014. We aimed to explore the relationship between serum ALP and the risk of depression in a large sample of adults in the general population and performed a multifaceted sensitivity analysis.

## 2. Materials and methods

### 2.1. Data sources

Our data were obtained from the National Health and Nutrition Examination Survey (NHANES), an ongoing series of sample surveys in which participants are selected for inclusion using a multistage, multistratified probabilistic approach (23), with the aim of collecting nationally representative data from the noninstitutionalized U.S. general population. The project conducted extensive household interviews to collect demographic baseline information and health questionnaire data. A mobile examination center (MEC) was used to perform physical examinations and collect blood samples. Serum samples were tested at the Laboratory Sciences Division of the National Center for Environmental Health at the Centers for Disease Control and Prevention. Detailed specimen collection and handling instructions are discussed in the NHANES Laboratory/Medical Technician Procedures Manual (LPM). Serum specimens were stored under appropriate frozen (−30°C) conditions until transported to the National Center for Environmental Health for testing. The following variables were included in our study: including demographic information, physical examination indicators, laboratory test results, health questionnaires, and the presence of comorbid hypertension and diabetes mellitus.

### 2.2. Study population

Our study design was a cross-sectional study, based on continuous publicly available data from NHANES 2007–2014, with all data details taken from the official NHANES website,^1^ which is freely available to all. Participants in our study were aged 18 years or older and completed interviews and assessments at the MEC. Data on depressive status, ALP and all covariates were excluded if they were missing. The final 17,485 participants (8,669 men and 8,816 women) participated in the retrospective study. The NHANES survey protocol was approved by the National Center for Health Statistics Research Ethics Review Committee and all participants provided written informed consent.

### 2.3. Depressive symptoms

All participants assessed depression status in NHANES using the Patient Health Questionnaire (PHQ-9), a nine-item screening instrument that measures the frequency of various depressive symptoms during the previous 2 weeks (24). Each of the nine items consists of responses on a four-point scale, with 0 = “not at all,” 1 = “a few days,” 2 = “more than half the days,” and 3 = “almost every day,” for a total score of 0–27. Participants were divided into a depressed group (≥10 scores) and a non-depressed group (<10 scores); additionally, participants were defined as depressed if they answered some questions on the PHQ-9 items but their total score was above 10. The PHQ-9 had a sensitivity of 88% and specificity of 88% for suggesting moderate to severe depressive symptoms (25).

### 2.4. Covariates

The NHANES database contains lifestyle information and personal medical history based on standardized questionnaires. Age, gender, race/ethnicity, education level, body mass index (BMI), the ratio of family income to poverty (PIR), smoking status, alcohol consumption status, physical activity, and laboratory findings including white blood cells (WBC), albumin (ALB), aspartate aminotransferase (AST), alanine aminotransferase (ALT), alkaline phosphatase (ALP), total cholesterol (TC), triglycerides (TG), creatinine, and the presence of comorbid diabetes and hypertension. Race was categorized as non-Hispanic white, non-Hispanic black, Mexican-American, other Hispanic, or other. Education level was categorized as less than high school, high school graduate, and college or higher. BMI was calculated as weight in kilograms divided by height in meters squared according to standardized protocols and was categorized as <25.0, 25.0 ~ 30.0, and ≥ 30.0 kg/m^2^. Smoking status was grouped into smokers and nonsmokers. All participants who had smoked more than 100 cigarettes during their lifetime were considered smokers. Participants who had not smoked even 100 cigarettes in their lifetime were considered nonsmokers. Participants who had had at least 12 drinks per year during their lifetime were considered drinkers. Physical activity was categorized into three intensity levels based on walking, moderate, and vigorous activity. Cases of diabetes were defined according to the American Diabetes Association criteria (26) and self-report questionnaires. The criteria were as follows: FPG ≥ 126 mg/dL, HbA1c ≥ 6.5%, 2-h plasma glucose from an oral glucose tolerance test ≥200 mg/dL, current use of insulin or diabetic pills to lower blood glucose, and self-reported questionnaire data indicating a physician diagnosis of diabetes. In addition, self-reported current use of antihypertensive medication or physician’s diagnosis was used to define hypertension.

### 2.5. Statistical analysis

Data analysis was performed using mean ± standard deviation (normal distribution), median ± interquartile range (IQR) (skewed distribution), and frequency (percentage) for demographic and clinical indicators, describing continuous and categorical variables, respectively. The *t*-test was used for group comparisons between normal distributions in continuous variables, the Kruskal-Wallis test for group comparisons between skewed distributions, and the chi-square test for categorical variables. The Kruskal-Wallis test or one-way ANOVA was applied to assess the significance of differences in groups grouped by ALP quartiles. To further analyze the relationship between different doses of ALP and the risk of depression, univariate and multivariate logistic regression were used. In multivariate logistic regression, we took logarithms for ALP when ALP was used as a continuous variable, and we used quartiles and a dichotomous model grouped by 79 U/L when ALP was used as a categorical variable. We show four different models: (1) unadjusted model, (2) model 1 adjusted for age and sex, (3) model 2 adjusted for age, sex, education level, PIR, race, BMI, smoking status, drinking status, and (4) model 3 adjusted for variables from model 2 and WBC, ALB, ALT, AST, TC, TG, creatinine, diabetes, hypertension. To identify modifications and interactions, we used a stratified logistic regression model and likelihood ratio test in subgroups of gender (female or male), age (<60 or ≥ 60 years), BMI (<25 kg/m^2^, 25 ~ 30 kg/m^2^, or ≥ 30 kg/m^2^), WBC (<10 × 10^9^/L or ≥ 10 × 10^9^/L), ALB (<40 g/L or ≥ 40 g/L), TG(<1.7 mmol/L or ≥ 1.7 mmol/L)and the status of smoking, diabetes mellitus and hypertension. All analyses were performed with the statistical package R (http://www.R-project.org, R Foundation) and the free statistical software version 1.5 (27). Statistical tests were two-sided and the significance level was set at *p* = 0.05.

## 3. Results

### 3.1. Study population

**Figure 1:**
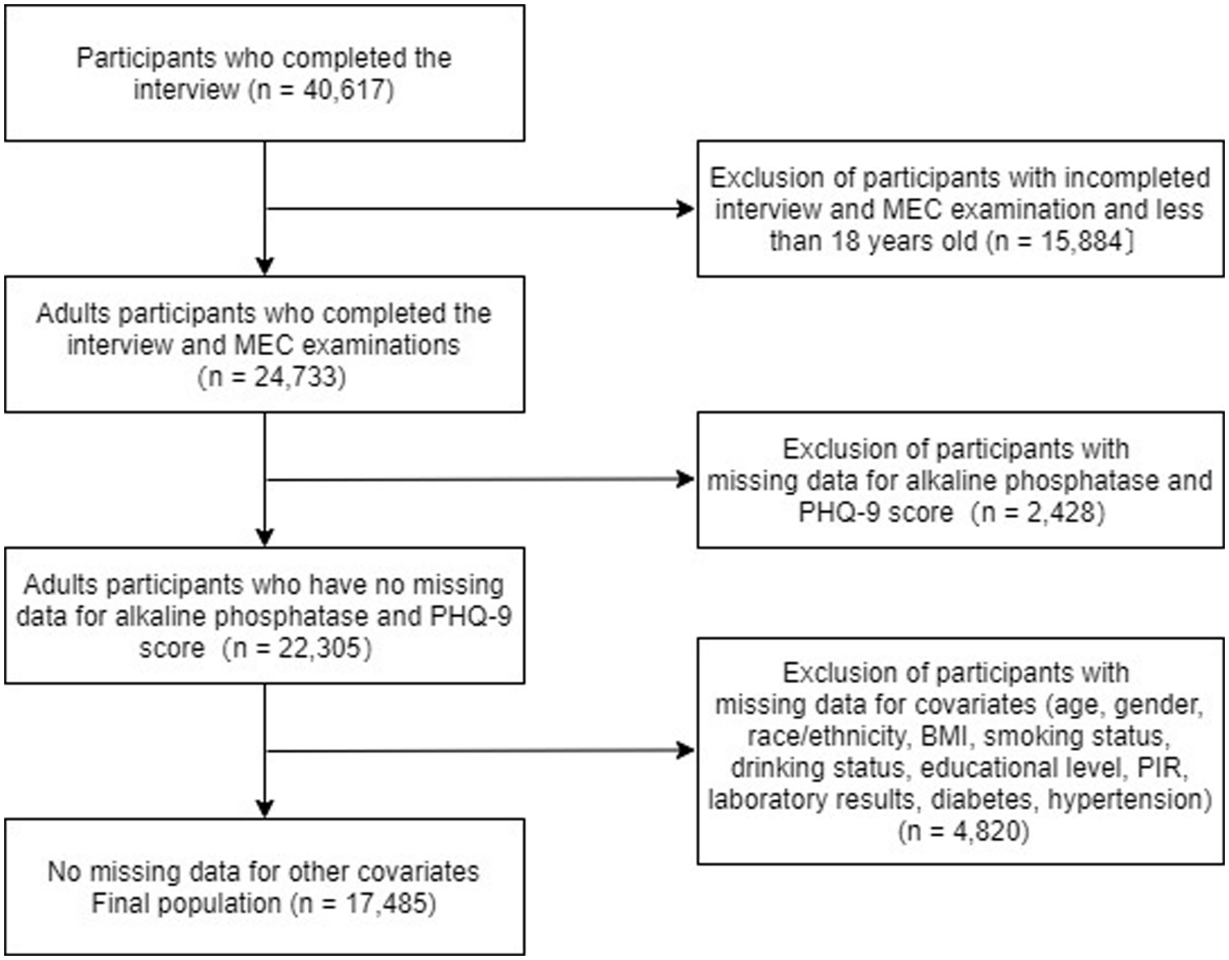
Participants inclusion flowchart.

This study used data from NHANES 2007–2014, from which we selected 40,617 potential participants, of which 24,733 adults (≥18 years) completed an interview and underwent MEC screening to be included in our study. Participants missing PHQ-9 scores and alkaline phosphatase (*n* = 2,428) were excluded. After excluding participants with missing covariate data (*n* = 4,820), the remaining 17,485 participants were included in our analysis. Figure 1 depicts a flow chart of the exclusion criteria.

### 3.2. Baseline characteristics

Table 1 shows the baseline characteristics of the study participants classified according to alkaline phosphatase quartiles. The mean age of the participants was 49.4 ± 17.7 years, of which 8,669 (49.6%) were male. The prevalence of depression was 9.3% (1,631/17,485) with a mean baseline ALP of 65.0 (53.0, 79.0). Participants with higher ALP levels were more likely to be older, less educated, and obese, and in addition, may have higher WBC, total cholesterol, TG, and concomitant diseases such as depression, hypertension, and diabetes. However, participants with higher ALP may have lower PIR and ALB levels. Interestingly, the higher the ALP, the higher the likelihood of smoking and, conversely, the lower the likelihood of drinking alcohol.

**Table 1:** Characteristics of the study participants by baseline serum alkaline phosphatase quartiles.

Covariates	Total (*n* = 17,485)	Quartile 1	Quartile 2	Quartile 3	Quartile 4	*P*
		≤ 52 U/L	53 ~ 64 U/L	65 ~ 78 U/L	≥ 79 U/L	
Age (years)	49.4 ± 17.7	46.0 ± 17.8	48.9 ± 17.6	49.7 ± 17.7	52.5 ± 17.0	<0.001
Gender						<0.001
Female	8,816 (50.4)	2,210 (54.1)	2,095 (47.3)	2,121 (48.5)	2,390 (51.9)	
Male	8,669 (49.6)	1,874 (45.9)	2,332 (52.7)	2,252 (51.5)	2,211 (48.1)	
Education level						<0.001
Did not graduate from high school	4,255 (24.3)	684 (16.7)	978 (22.1)	1,136 (26)	1,457 (31.7)	
Graduated from high school	3,988 (22.8)	825 (20.2)	1,012 (22.9)	1,030 (23.6)	1,121 (24.4)	
College education or above	9,242 (52.9)	2,575 (63.1)	2,437 (55)	2,207 (50.5)	2,023 (44)	
Race/ethnicity						<0.001
Mexican American	2,518 (14.4)	335 (8.2)	573 (12.9)	666 (15.2)	944 (20.5)	
Other Hispanic	1,674 (9.6)	296 (7.2)	381 (8.6)	440 (10.1)	557 (12.1)	
Non-Hispanic white	8,217 (47.0)	2,155 (52.8)	2,125 (48)	2,071 (47.4)	1,866 (40.6)	
Non-Hispanic black	3,477 (19.9)	782 (19.1)	871 (19.7)	848 (19.4)	976 (21.2)	
Other races	1,599 (9.1)	516 (12.6)	477 (10.8)	348 (8)	258 (5.6)	
PIR	2.5 ± 1.6	2.8 ± 1.7	2.6 ± 1.7	2.4 ± 1.6	2.3 ± 1.6	<0.001
BMI (kg/m^2^)	29.1 ± 6.9	27.7 ± 6.5	28.8 ± 6.6	29.4 ± 6.8	30.4 ± 7.2	<0.001
Smoking status						<0.001
Smoker	8,018 (45.9)	1,675 (41)	2,033 (45.9)	2,029 (46.4)	2,281 (49.6)	
Non-smoker	9,467 (54.1)	2,409 (59)	2,394 (54.1)	2,344 (53.6)	2,320 (50.4)	
Drinking status						<0.001
Drinker	12,771 (73.0)	3,138 (76.8)	3,340 (75.4)	3,162 (72.3)	3,131 (68.1)	
Non-drinker	4,714 (27.0)	946 (23.2)	1,087 (24.6)	1,211 (27.7)	1,470 (31.9)	
Physical activity						
Vigorous work activity	14,207 (81.3)	3,390 (83)	3,552 (80.3)	3,483 (79.6)	3,782 (82.2)	<0.001
Moderate work activity	11,142 (63.7)	2,600 (63.7)	2,781 (62.8)	2,726 (62.3)	3,035 (66)	0.002
Walk or bicycle	12,929 (73.9)	2,945 (72.1)	3,245 (73.3)	3,235 (74)	3,504 (76.2)	<0.001
Vigorous recreational activities	13,776 (78.8)	2,946 (72.1)	3,426 (77.4)	3,487 (79.7)	3,917 (85.1)	<0.001
Moderate recreational activities	10,356 (59.2)	2,112 (51.7)	2,534 (57.3)	2,662 (60.9)	3,048 (66.2)	<0.001
Laboratory results						
WBC (×10^9^/L)	7.2 ± 2.5	6.8 ± 1.9	7.0 ± 2.0	7.3 ± 2.4	7.7 ± 3.2	<0.001
ALB (g/L)	42.4 ± 3.4	43.0 ± 3.2	42.7 ± 3.2	42.5 ± 3.3	41.7 ± 3.6	<0.001
ALT (U/L)	21.0 (16.0, 28.0)	19.0 (15.0, 25.0)	21.0 (16.0, 28.0)	21.0 (17.0, 29.0)	23.0 (17.0, 31.0)	<0.001
AST (U/L)	23.0 (20.0, 28.0)	22.0 (19.0, 26.0)	23.0 (20.0, 27.0)	23.0 (20.0, 28.0)	24.0 (20.0, 30.0)	<0.001
ALP (U/L)	65.0 (53.0, 79.0)	46.0 (41.0, 49.0)	59.0 (56.0, 61.0)	71.0 (67.0, 74.0)	91.0 (84.0, 103.0)	<0.001
TC (mmol/L)	5.0 ± 1.1	4.9 ± 1.0	5.0 ± 1.1	5.1 ± 1.1	5.1 ± 1.2	<0.001
TG (mmol/L)	1.4 (0.9, 2.1)	1.2 (0.8, 1.8)	1.3 (0.9, 2.1)	1.4 (1.0, 2.2)	1.6 (1.0, 2.4)	<0.001
Creatinine (μmol/L)	75.1 (63.6, 89.3)	74.3 (63.6, 89.3)	76.0 (64.5, 90.2)	76.0 (63.6, 89.3)	74.3 (63.6, 89.3)	0.009
Depression	1,631 (9.3)	279 (6.8)	379 (8.6)	428 (9.8)	545 (11.8)	<0.001
Diabetes	3,139 (18.0)	569 (13.9)	696 (15.7)	745 (17)	1,129 (24.5)	<0.001
Hypertension	6,320 (36.1)	1,203 (29.5)	1,533 (34.6)	1,604 (36.7)	1,980 (43)	<0.001

Data were mean ± SD or median (IQR) for continuous variables or numbers (proportions) for categorical variables. PIR, the ratio of family income to poverty; BMI, body mass index; WBC, white blood cell; ALB, albumin; ALT, alanine aminotransferase; AST, aspartate aminotransferase; ALP, alkaline phosphatase; TC, total cholesterol; TG, triglyceride; Q1, Q2, Q3, and Q4 are quartiles of ALP.

### 3.3. Univariate and multivariate analyses between alkaline phosphatase and depression

In Table 2, serum ALP was calculated as a continuous variable, showing that age, gender, education level, PIR, BMI, smoking status, WBC, albumin, AST, ALP, TG, comorbid diabetes, and hypertension were significantly associated with the risk of depression. Table 3 summarizes the ORs and corresponding 95% CIs for the risk of depression according to serum ALP (log_2_) and ALP quartiles and LDH ≥79 U/L. After adjustment for different confounders, the ORs for serum ALP were consistently significant in all three models, regardless of ALP as a continuous variable (log_2_) or quartile (OR range 1.19–1.34, *p* < 0.05) or dichotomous classification (≥79 U/L). Serum ALP was assessed as a continuous variable (log_2_), the full variable adjusted model (model 3), with an OR of 1.19 (95% CI: 1.05–1.34). ALP analysis as quartiles, also in model 3, the adjusted OR for depression in Q2, Q3 and Q4 was 1.21 (95% CI: 1.02–1.43), 1.26 (95% CI: 1.06–1.48), and 1.34 (95% CI: 1.14–1.58), respectively, using Q1 as a reference. In the ALP dichotomous variable, the OR of serum ALP with depression was 1.14 (95% CI: 1.02–1.29) for ≥79 U/L compared to <79 U/L. Furthermore, significant in all models (Table 3, trend *p* < 0.05), indicating that serum ALP was positively associated with depression.

**Table 2:** Association of covariates and risk of depression.

Variable	OR 95% CI	*p*-value
Age (years)	1.00 (0.99 ~ 1.00)	0.025
Gender		
Female	1.00 (reference)	
Male	0.52 (0.46 ~ 0.57)	<0.001
Education level		
Did not graduate from high school	1.00 (reference)	
Graduated from high school	0.66 (0.58 ~ 0.76)	<0.001
College education or above	0.47 (0.41 ~ 0.52)	<0.001
Race/ethnicity		
Mexican American	1.00 (reference)	
Other Hispanic	1.42 (1.17 ~ 1.73)	<0.001
Non-Hispanic white	0.95 (0.82 ~ 1.11)	0.550
Non-Hispanic black	1.05 (0.88 ~ 1.25)	0.599
Other races	0.66 (0.52 ~ 0.84)	0.001
PIR	0.66 (0.63 ~ 0.68)	<0.001
BMI (kg/m^2^)	1.04 (1.03 ~ 1.05)	<0.001
Smoking status		
Smoker	1.00 (reference)	
Non-smoker	0.53 (0.48 ~ 0.59)	<0.001
Drinking status		
Drinker	1.00 (reference)	
Non-drinker	1.00 (0.89 ~ 1.12)	0.988
Laboratory results		
WBC (×10^9^/L)	1.07 (1.05 ~ 1.09)	<0.001
ALB (g/L)	0.93 (0.91 ~ 0.94)	<0.001
ALT (U/L)	1.00 (1.00 ~ 1.00)	0.055
AST (U/L)	1.00 (1.00 ~ 1.00)	0.025
ALP (log_2_) (U/L)	1.62 (1.45 ~ 1.81)	<0.001
TC (mmol/L)	1.05 (1.00 ~ 1.10)	0.053
TG (mmol/L)	1.08 (1.05 ~ 1.12)	<0.001
Creatinine (μmol/L)	1.00 (1.00 ~ 1.00)	0.973
Physical activity		
Vigorous work activity	1.07 (0.94 ~ 1.22)	0.327
Moderate work activity	1.24 (1.11 ~ 1.38)	<0.001
Walk or bicycle	1.22 (1.08 ~ 1.37)	0.001
Vigorous recreational activities	2.54 (2.15 ~ 2.99)	<0.001
Moderate recreational activities	2.24 (1.99 ~ 2.51)	<0.001
Diabetes	1.69 (1.50 ~ 1.90)	<0.001
Hypertension	1.64 (1.48 ~ 1.82)	<0.001

Data presented are ORs and 95% CIs. PIR, the ratio of family income to poverty; BMI, body mass index; WBC, white blood cell; ALB, albumin; ALT, alanine aminotransferase; AST, aspartate aminotransferase; ALP, alkaline phosphatase; TC, total cholesterol; TG, triglyceride.

**Table 3:** The association between baseline alkaline phosphatase and the risk of depression.

Variable (U/L)	Non-adjusted		Model 1		Model 2		Model 3	
	OR 95%CI	*P*-value	OR 95%CI	*P*-value	OR 95%CI	*P*-value	OR 95%CI	*P*-value
Log_2_ALP	1.62 (1.45 ~ 1.82)	<0.001	1.68 (1.50 ~ 1.88)	<0.001	1.31 (1.16 ~ 1.48)	<0.001	1.20 (1.06 ~ 1.36)	0.004
Quartiles								
Q1(≤52)	Ref.		Ref.		Ref.		Ref.	
Q2(53 ~ 64)	1.28 (1.09 ~ 1.50)	0.003	1.36 (1.16 ~ 1.600)	<0.001	1.21 (1.03 ~ 1.43)	0.024	1.20 (1.02 ~ 1.42)	0.031
Q3(65 ~ 78)	1.48 (1.26 ~ 1.73)	<0.001	1.58 (1.35 ~ 1.85)	<0.001	1.28 (1.09 ~ 1.51)	0.003	1.25 (1.06 ~ 1.47)	0.008
Q4(≥79)	1.84 (1.58 ~ 2.14)	<0.001	1.95 (1.67 ~ 2.28)	<0.001	1.46 (1.24 ~ 1.71)	<0.001	1.34 (1.14 ~ 1.58)	<0.001
*P* for trend		<0.001		<0.001		<0.001		0.001
Categories								
ALP < 79	Ref.		Ref.		Ref.		Ref.	
ALP ≥ 79	1.47 (1.31 ~ 1.63)	<0.001	1.48 (1.33 ~ 1.66)	<0.001	1.24 (1.10 ~ 1.39)	<0.001	1.15 (1.02 ~ 1.29)	0.020

Model 1: adjusted for age and gender. Model 2: adjusted for the variables in Model 1 plus education level, PIR, race, BMI, smoking status, and drinking status. Model 3: adjusted for the variables in Model 2 plus WBC, ALB, ALT, AST, TC, TG, diabetes, and hypertension.

### 3.4. Subgroup analyses by adjusted potential effect confounders

**Figure 2:**
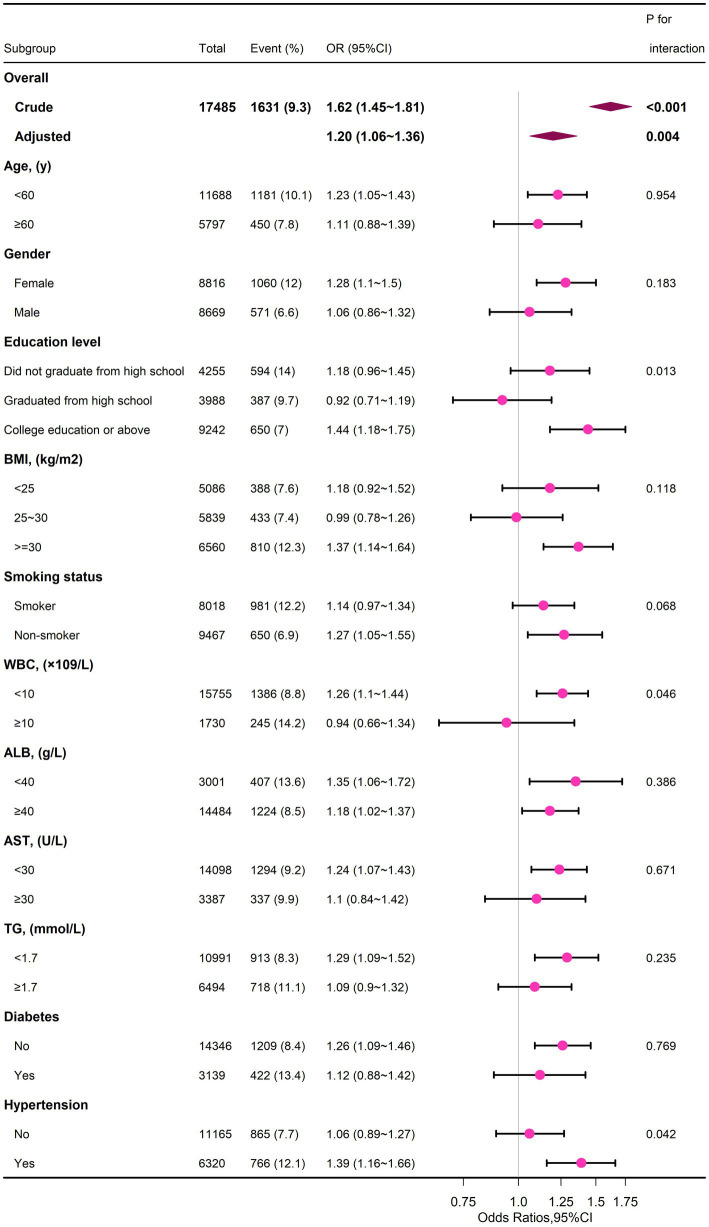
Subgroup analyses of the serum ALP and depression.

To test whether there was an association between serum ALP levels and the risk of depression in different subgroups, stratified and interaction analyses (selected if *p* value <0.05) were performed based on the results of univariate analyses, selected on age (<60 or ≥ 60 years), gender (male or female), education level (did not graduate from high school, graduated from high school, or college education or above), BMI (<25.0, 25.0–29.9, or ≥ 30.0 kg/m^2^), smoking status (smoker or non-smoker), WBC (<10, or ≥ 10 × 10^9^/L), albumin (<40 or ≥ 40 g /L), AST (<30 or ≥ 30 U/L), TG (<1.7 or ≥ 1.7 mmol/L), diabetes mellitus (yes or no), and hypertension (yes or no) in the assessment of the effect of serum ALP (log_2_) (per 1-unit increment) on the risk of depression in different subgroups. In the association between serum ALP and risk of depression, no variables played an interactive role except education level, WBC, and comorbid hypertension (*p* > 0.05 for interaction). This subgroup analysis was adjusted for age, sex, race, education level, PIR, BMI, smoking status, drinking status, WBC, ALB, ALT, AST, TC, TG, diabetes, and hypertension, consistent with Model 3 (Supplementary Table S1), and the forest plot drawn is shown in Figure 2.

## 4. Discussion

Our study found that in a large survey of adults, participants with higher serum ALP, even within the normal range, had a significantly increased risk of depression. The present study was adjusted for the covariates/confounders considered to fully explore the association between serum ALP and risk of depression in adults, and the findings suggest that the association between serum ALP and depression was consistent across these subgroups: age (<60, or ≥ 60 years), gender (male or female), BMI (<25.0, 25.0–29.9, or ≥ 30.0 kg/m^2^), smoking status (smoker or non-smoker), albumin (<40, or ≥ 40 g/L), AST (<30 or ≥ 30 U/L), TG (<1.7, or ≥ 1.7 mmol/L), and diabetes (yes or no).

Serum alkaline phosphatase levels may vary by gender (28). Li et al. (21) noted that the correlation between alkaline phosphatase levels and increased risk of depression differed between men and women. The bone rebuilding process is increased in postmenopausal women due to estrogen deficiency, and the bone rebuilding process can be regulated by ALP levels (29). We therefore hypothesized that an accelerated bone rebuilding process may further increase the risk of depression. However, in our subgroup analysis, we did not find an effect of ALP on the increased risk of depression in patients stratified by gender. It is possible that we adjusted for important covariates such as biochemical indicators and co-morbidities (presence of comorbid diabetes and hypertension). Of course, further prospective studies are needed to confirm this finding in men.

According to Pascoe MC, reduced serum albumin levels after stroke were associated with long-term depressive symptoms in elderly Swedish patients (30), which is consistent with the results in our univariate analysis, so in our study, adjusted for albumin levels, we found no significant effect of albumin levels on the relationship between ALP and depression risk. Also, triglycerides were shown to be negatively associated with the risk of depression in univariate analysis, which is also consistent with previous studies (31), and showed no effect on the significant association between ALP and the risk of depression after adjusting for TG. In our study, it was shown that ALP was significantly associated with the risk of depression in those with higher levels of education. In addition, the relationship did not seem to be significant in those with WBC ≥10 × 10^9^/L, which may be due to the distribution of the sample size. The association between ALP and risk of depression is more readily observed in hypertensive populations, and previous studies in hypertensive individuals have shown that higher serum ALP increases the risk of endothelial dysfunction (32). Larger prospective studies are needed to confirm these findings.

The exact mechanism by which high serum alkaline phosphatase is associated with high risk of depression remains to be elucidated. However, our findings are biologically plausible based on the available evidence. ALP is a cell surface protein that exists in soluble form in plasma and other body fluids, and TNAP is expressed in the brain, endothelial cells, neuronal membranes and synaptic gaps (14). Therefore, changes in cerebrospinal fluid (33) and plasma (34) ALP activity may be the result of central nervous system damage. Hypophosphatasia (HPP) is a rare genetic metabolic disorder with mutations in the ALPL gene, which, in addition to causing problems with bone and tooth mineralization, tends to produce problems associated with the central nervous system (CNS), such as seizures, anxiety and depression. The same was reported in a study of neo-coronary pneumonia (18). This is probably because ALP is present on neuronal membranes and increases with brain injury and cerebrovascular disease—suggesting that increased ALP is associated with neuronal loss (35). Due to the role of ALP in γ-aminobutyric acid (GABA) metabolism (36), this neuron is thought to play a role in developmental plasticity and activity-dependent cortical function (14, 37). Additionally, elevated ALP may also be caused by antidepressants (38).

In this study, we found that the risk of depression increased with increasing ALP levels, even within the normal range, and the serum ALP was a risk factor for depression (*p* < 0.05). This study has several strengths. First, we used a large, nationally representative sample of US adults. Second, we included and adjusted for known and potential risk factors for depressive symptoms. Third, this study examined the association of serum ALP as a continuous variable, categorized by quartiles, and dichotomized by 79 U/L with depression. In addition, we performed sensitivity analyses to assess the effect of the correlation between ALP and depression risk across subgroups.

However, the detailed biological underlying mechanisms remain to be further investigated. Limitations of the current study should also be noticed. First, the present study is a cross-sectional one and causal inference is not possible. Second, we cannot rule out the possibility that the observed association is due to unmeasured residual confounding, although we have adjusted for a wide range of covariates in the regression model. Third, the NHANES project is based on Americans, and some variables are based on self-reporting; therefore, misinterpretation of the questions or recall issues may arise, while generalization to other populations requires further validation. Fourth, it is possible that tests of laboratory indicators may be influenced by status, such as diet and antidepressants. Fifth, in our current study, the majority of participants had normal ALP levels and we were unable to investigate the association with relatively very low or very high ALP levels with increased risk of depression. Finally, in the present study, we measured serum ALP at baseline, and in the future, more frequent measurement of ALP levels would allow a more accurate assessment of its progression over time. Because NHANES participants are selected using a multi-stage, multi-stratified probabilistic design, examining a large population each year avoids selection bias to some extent. Nevertheless, given these limitations, well-designed multicenter randomized controlled trials are essential to validate our findings.

In summary, in this cross-sectional study, we found that the risk of depression in US adults increased with rising serum ALP levels, even within the normal range. If our findings are further confirmed, serum ALP levels may be a valuable additional reference biomarker for assessing the risk of depression.

## Data availability statement

The raw data supporting the conclusions of this article will be made available by the authors, without undue reservation.

## Ethics statement

The NHANES survey protocol was approved by the Institutional Research Ethics Review Board of the CDC National Center for Health Statistics. All participants provided written informed consent, and the study was approved by the National Center for Health Statistics Research Ethics Review Committee (https://wwwn.cdc.gov/nchs/nhanes/default.aspx).

## Author contributions

YL conceived the study hypothesis. WL and MS performed the data analysis. LL and JX drafted the manuscript. HY and YM revised it critically for important intellectual content, and supervised the writing of the manuscript. All authors contributed to the article and approved the submitted version.

## Conflict of interest

The authors declare that the research was conducted in the absence of any commercial or financial relationships that could be construed as a potential conflict of interest.

## Publisher’s note

All claims expressed in this article are solely those of the authors and do not necessarily represent those of their affiliated organizations, or those of the publisher, the editors and the reviewers. Any product that may be evaluated in this article, or claim that may be made by its manufacturer, is not guaranteed or endorsed by the publisher.

## Footnotes

^1^ https://www.cdc.gov/nchs/nhanes/about_nhanes.htm
