# Supplementary material for: Association of serum alkaline phosphatase and depression in US adults: a population-based cross-sectional study
Source: Front Psychiatry. 2023 May 17;14:1131105. doi: 10.3389/fpsyt.2023.1131105 (PMC10229779; doi:10.3389/fpsyt.2023.1131105)
Supplement: Supplementary file 1 [file Table_1.DOCX]

Supplementary table S1. Subgroup analyses of the serum ALP and depression.

| **Subgroup** | **Total** | **Event (%)** | **OR (95%CI)** | **P for interaction** |
| --- | --- | --- | --- | --- |
| **Overall** |  |  |  |  |
| **Crude** | 17485 | 1631 (9.3) | 1.62 (1.45~1.81) | <0.001 |
| **Adjusted** |  |  | 1.20 (1.06~1.36) | 0.004 |
| **Age, (y)** |  |  |  |  |
| <60 | 11688 | 1181 (10.1) | 1.23 (1.05~1.43) | 0.954 |
| ≥60 | 5797 | 450 (7.8) | 1.11 (0.88~1.39) |  |
| **Gender** |  |  |  |  |
| Female | 8816 | 1060 (12) | 1.28 (1.1~1.5) | 0.183 |
| Male | 8669 | 571 (6.6) | 1.06 (0.86~1.32) |  |
| **Education level** |  |  |  |  |
| Did not graduate from high school | 4255 | 594 (14) | 1.18 (0.96~1.45) | 0.013 |
| Graduated from high school | 3988 | 387 (9.7) | 0.92 (0.71~1.19) |  |
| College education or above | 9242 | 650 (7) | 1.44 (1.18~1.75) |  |
| **BMI, (kg/m^2^)** |  |  |  |  |
| <25 | 5086 | 388 (7.6) | 1.18 (0.92~1.52) | 0.118 |
| 25~30 | 5839 | 433 (7.4) | 0.99 (0.78~1.26) |  |
| ≥30 | 6560 | 810 (12.3) | 1.37 (1.14~1.64) |  |
| **Smoking status** |  |  |  |  |
| Smoker | 8018 | 981 (12.2) | 1.14 (0.97~1.34) | 0.068 |
| Non-smoker | 9467 | 650 (6.9) | 1.27 (1.05~1.55) |  |
| **WBC, (×10^9^/L)** |  |  |  |  |
| <10 | 15755 | 1386 (8.8) | 1.26 (1.1~1.44) | 0.046 |
| ≥10 | 1730 | 245 (14.2) | 0.94 (0.66~1.34) |  |
| **ALB, (g/L)** |  |  |  |  |
| <40 | 3001 | 407 (13.6) | 1.35 (1.06~1.72) | 0.386 |
| ≥40 | 14484 | 1224 (8.5) | 1.18 (1.02~1.37) |  |
| **AST, (U/L)** |  |  |  |  |
| <30 | 14098 | 1294 (9.2) | 1.24 (1.07~1.43) | 0.671 |
| ≥30 | 3387 | 337 (9.9) | 1.1 (0.84~1.42) |  |
| **TG, (mmol/L)** |  |  |  |  |
| <1.7 | 10991 | 913 (8.3) | 1.29 (1.09~1.52) | 0.235 |
| ≥1.7 | 6494 | 718 (11.1) | 1.09 (0.9~1.32) |  |
| **Diabetes** |  |  |  |  |
| No | 14346 | 1209 (8.4) | 1.26 (1.09~1.46) | 0.769 |
| Yes | 3139 | 422 (13.4) | 1.12 (0.88~1.42) |  |
| **Hypertension** |  |  |  |  |
| No | 11165 | 865 (7.7) | 1.06 (0.89~1.27) | 0.042 |
| Yes | 6320 | 766 (12.1) | 1.39 (1.16~1.66) |  |
